# A Respiratory Sensor-Based Study of the Relationship between Voluntary Breathing Patterns and Aerobic and Anerobic Exercise Capacity—An Exploratory Applied Study

**DOI:** 10.3390/s24196310

**Published:** 2024-09-29

**Authors:** Yinling Du, Kai Jiang, Haojie Li

**Affiliations:** 1 Department of Physical Education, Hangzhou Normal University, Hangzhou 311121, China; 20200021@hznu.edu.cn; 2 Department of Public Sports and Arts, Zhejiang University, Hangzhou 310058, China; 3 School of Exercise and Health, Shanghai University of Sport, Shanghai 200438, China; 202121070037@mail.bnu.edu.cn

**Keywords:** respiratory patterns, inhalation/exhalation ratio, 50 m sprint, 800 m run, 12 min run, aerobic and anerobic exercise capacities

## Abstract

(1) Background: Exploring the relationship between spontaneous breathing patterns and aerobic and anerobic running exercise performance can greatly improve our understanding of optimizing physical fitness. Spontaneous breathing patterns refer to how the rhythm and depth of breathing affect performance and physical adaptation during exercise. (2) Methods: This study aimed to investigate this relationship by enrolling 240 college students (120 males and 120 females, aged 18–22). We evaluated their resting respiratory rate (RR), the combined total of abdominal and thoracic movements (AM+TM), the proportion of abdominal movement to the overall respiratory movement (AM/(AM+TM)), and the inhalation to exhalation ratio (I/E ratio). Additionally, their performance in a 50 m sprint (measuring anerobic capability), an 800- or 1000 m run (assessing mixed aerobic and anerobic capacity), and a 12 min distance run (evaluating aerobic capacity) was recorded. (3) Results: Our findings, through both correlational and comparative analyses, indicate that a larger AM+TM is predictive of a greater distance covered in the 12 min run, suggesting enhanced aerobic capacity. Interestingly, among female participants, a lower body mass index (BMI) coupled with a higher proportion of abdominal movement (AM/(AM+TM)) was linked to better performance in the 800 m run, indicative of superior mixed aerobic and anerobic capacities. These results imply that women with a larger tidal volume and those with a lower BMI but higher abdominal contribution to breathing at rest may exhibit better aerobic and mixed exercise capacities, respectively. (4) Conclusions: Based on these findings, we recommend that healthcare professionals and physical education instructors incorporate respiratory pattern assessments into their practices to potentially improve the physical health of their clients and students, with a particular emphasis on female populations. This study underscores the importance of understanding the intricate relationship between spontaneous respiratory patterns and exercise capabilities in enhancing overall physical fitness and health.

## 1. Introduction

Among the top 20 global risk factors, high blood pressure, elevated body mass index (BMI), and increased fasting plasma glucose levels are identified as the principal metabolic risks [1]. It has been reported that approximately 22% of US adults manifest the metabolic syndrome [2], spotlighting metabolic health as a focal point of scientific interest [3]. Within this context, as an important component of metabolic health, enhancing aerobic and anerobic metabolic capacities emerges as a pivotal health intervention strategy [4]. Aerobic capacity refers to the ability of large muscle groups to perform work over extended periods through energy produced via aerobic metabolism, encompassing activities such as cycling, dancing, hiking, jogging or long-distance running, swimming, and walking [5]. The American College of Sports Medicine (ACSM) describes aerobic capacity as the combined ability of the cardiorespiratory system to deliver oxygen and of skeletal muscles to utilize it [6]. Previous studies have shown that aerobic exercises can help people burn calories, improve cardiovascular health, boost mental well-being [7,8,9], and reduce the risk of developing metabolic syndrome or diabetes [10,11], as well as cardiac events [12]. The key indicator to measure it is the peak oxygen consumption (VO2 max) [5], which is intricately linked to both all-cause and cardiovascular-specific mortality in individuals with coronary heart disease. Notably, each 1 mL·kg^−1^·min^−1^ increase in VO2 max corresponds to an approximately 15% reduction in mortality risk [13]. Contrastingly, anerobic capacity is the muscle’s capability to support high-intensity, short-duration activities [14], such as sprinting, weightlifting, and long jumping, primarily fueled by the ATP-CP system and anerobic glycolysis [15]. Anerobic exercise is lauded for its benefits to bone health and the cardiovascular system [5], along with its capacity to bolster muscular strength and endurance [16,17].

Respiratory activity is crucial for both aerobic and anerobic energy production, with breathing patterns adjusting to the specific demands of each metabolic state. Under aerobic conditions, sustained deep breathing is essential to ensure an adequate oxygen supply. In contrast, anerobic activities require rapid, shallow breaths to efficiently expel metabolic by-products such as carbon dioxide and lactic acid [18]. Key aspects of breathing patterns include the respiration rate (RR), breath depth, the inhalation-to-exhalation ratio (I/E ratio), the sequence of chest wall movements during breathing, the utilization of accessory muscles, and breath symmetry [19]. Research indicates that RR significantly affects muscle strength, oxygen saturation, carbon dioxide partial pressure, autonomic nervous system activity, and cardiovascular system performance [19,20,21,22]. Furthermore, a lower RR has been associated with decreased basal heart rates and blood pressure, improved oxygenation, and enhanced baroreflex sensitivity and function [23,24]. The I/E ratio, which represents the duration of inspiration relative to expiration, is also significant. Statistically, this ratio averages 1:1.21 for men and 1:1.14 for women, with no significant gender difference [25]. Additionally, the I/E ratio has a substantial impact on autonomic nervous system activity [26,27], which may, in turn, indirectly affect physical performance.

Respiratory measurements play an important role in the development of athletic training programs, especially in the physical fitness management of college students, providing athletes and coaches with critical information about an individual’s physiological state and athletic ability. With the development of exercise science, more and more studies have focused on the relationship between respiratory patterns and athletic performance, showing that respiration is not only a fundamental physiological process for sustaining life but also influences endurance, strength, and overall performance during exercise [28]. First, respiratory rate and depth directly affect oxygen uptake and carbon dioxide excretion, which are critical for energy supply during exercise. Studies have shown that appropriate breathing patterns can increase the efficiency of oxygen utilization during exercise, thereby enhancing performance [29]. Previous research has found that deep, slow breathing significantly improves athletes’ performance in endurance sports. This finding highlights the importance of incorporating breathing exercises in training to optimize physiological adaptations in athletes, especially in the college population, where good breathing patterns can help improve their athletic performance and physical fitness [30]. Secondly, respiratory measurements help to identify the level of fatigue and recovery state of athletes. Monitoring changes in respiratory rate and pattern allows coaches to assess the physiological load of athletes in real time, thus adjusting the intensity and content of training. It has also been found that changes in the respiratory rate of athletes after high-intensity training can reflect their fatigue level, and timely respiratory monitoring can help to develop a personalized recovery plan to avoid overtraining and potential sports injuries. This adjustment strategy based on physiological data improves the science and effectiveness of training, especially for the college population, which can effectively prevent sports injuries and promote health [31]. Therefore, respiratory measurement has important scientific value and application prospects in sports training programs. By monitoring and analyzing respiratory patterns, coaches can better understand the physiological state of college students, develop personalized training programs, improve athletic performance, and promote physical health. Therefore, future research should continue to explore the application of respiratory measurement in athletic training to promote the development of exercise science and the physical health of college students.

Respiratory monitoring techniques have undergone significant development in past research, employing a variety of sensors to assess breathing patterns and their effects on exercise performance. Traditional respiratory monitoring methods mainly include airflow sensors, pressure sensors, and infrared sensors [32]. For example, it has been shown that airflow sensors assess respiratory rate and depth by measuring changes in the velocity and volume of the respiratory airflow and are commonly used in clinical settings to monitor the respiratory status of patients. However, the applicability of this method in dynamic exercise environments is limited because airflow changes during exercise may lead to inaccurate data [33]. Pressure sensors, on the other hand, infer respiratory patterns by monitoring pressure changes in the thoracic and abdominal cavities. This approach has been used in certain studies, such as in the field of exercise physiology, where researchers have used pressure sensors to analyze respiratory changes in athletes under different intensities of training. However, the use of pressure sensors often requires complex equipment setups and can be interfered with by external factors during exercise, affecting the reliability of the data [34]. There is also research evidence that infrared sensors, on the other hand, assess respiratory rate by measuring changes in heat generated during respiration, and while this method performs well in some situations, its sensitivity to ambient temperature and humidity limits its application in variable exercise environments. Despite the achievements of the above sensors in respiration monitoring, there are still some shortcomings [35]. First, many traditional sensors are less accurate and reliable in dynamic exercise environments, making it difficult to fully reflect changes in breathing patterns during exercise. Second, traditional methods often require more complex equipment setups, limiting their application in practical exercise training.

Therefore, in this study, we chose to use a respiratory rate (RR) sensor, which is an innovative technology based on resistive retractive sensors that can monitor participants’ respiratory rate and breathing patterns in real time and accurately. Compared to conventional sensors, RR sensors are more flexible and adaptable, providing reliable data under different exercise states. In addition, the use of RR sensors simplifies equipment setup, making respiratory monitoring possible during athletic training and competition. By employing the RR sensor, we were able to delve deeper into the relationship between spontaneous breathing patterns and aerobic and anerobic exercise capacity, thus providing a scientific basis for optimizing athletic performance and improving physical adaptations. The innovative and effective nature of this method makes it ideal for this study, overcoming the problems of data inaccuracy and equipment complexity that have characterized previous studies and providing a new perspective on the development of exercise physiology.

Thoracic and abdominal breathing represent two distinct breathing techniques. Abdominal breathing, characterized by slow, deep breaths, enhances oxygen uptake, thereby potentially improving aerobic capacity. This technique facilitates a more efficient use of the diaphragm, allowing for a greater volume of air with each breath, which is beneficial for aerobic activities [36]. In contrast, thoracic breathing involves faster, shallower breaths that can expedite oxygen intake and reduce lactate accumulation, thus effectively enhancing anerobic capacity [37]. This form of breathing is more common during high-intensity activities where quick oxygen delivery is crucial. Research indicates that breathing exercises, particularly those affecting intra-abdominal pressure, can bolster balance and postural stability [38]. For example, studies have identified a positive link between the extent of diaphragmatic movement during natural breathing and balance performance [39]. Specifically, employing abdominal drawing-in techniques during exercises such as squats has been shown to increase the activation of key muscles such as the rectus abdominis, biceps femoris, and tibialis anterior, leading to improved movement stability [40]. Furthermore, in women, respiratory movements have been observed to correlate positively, albeit weakly, with vertical jump performance, with a notable relationship between the extent of abdominal movement and overall motion (Rho = 0.39) [41].

Despite these insights, the direct relationship between spontaneous breathing patterns and aerobic versus anerobic capacities remains largely unexplored. Thus, this study seeks to delve into this area, aiming to uncover novel physiological markers that could aid in the assessment of physical health and inform training strategies.

## 2. Participants and Methods

### 2.1. Participants

This study recruited 240 college students (120 males and 120 females, aged 18–22) via random cluster sampling. A total of 240 participants were recruited for this study, 120 males and 120 females, all of whom were college students between the ages of 18 and 22. The mean age of the male participants was 20.1 years (standard deviation 1.2) and the mean age of the female participants was 19.9 years (standard deviation 1.1). In terms of body measurements, the mean height for males was 175.4 cm (standard deviation 6.3), weight was 70.2 kg (standard deviation 8.5), and BMI (body mass index) was 22.8 (standard deviation 2.5). Females had a mean height of 162.3 cm (standard deviation 5.8), weight of 55.8 kg (standard deviation 6.7), and BMI of 21.2 (standard deviation 1.9). Participants were generally more experienced in sports, with a mean of 3.5 years (standard deviation 1.8) for men and 3.2 years (standard deviation 1.5) for women. In addition, the mean number of sports participated in was 4.1 (standard deviation 1.2) for male participants and 3.8 (standard deviation 1.0) for females. These characteristics suggest that the participants were somewhat diverse in terms of physical fitness and athletic experience, providing a good basis for the study. The sample size was calculated based on a multiple regression analysis, utilizing G*Power to account for an anticipated 20% dropout rate, necessitating at least 120 participants. Considering a medium effect size (F2 = 0.15), an alpha level of 0.05, and a desired power of 0.90, with three predictors (age, BMI, and one respiratory pattern parameter) included in the analysis, the required sample size was determined to be 99 participants. To ensure a comprehensive analysis that accounts for significant physiological differences between genders, the sample size was adjusted to 240 participants, encompassing both males and females. The study was carried out in strict accordance with the principles of the Declaration of Helsinki and received ethical approval from the Research Ethics Committee of Zhejiang University (Approval Number: 2023–039). Prior to participation, all individuals provided written informed consent, affirming their voluntary participation and understanding of the study’s procedures and objectives. Because the university physical education program was fixed, all participants exercised only in the university selected physical education program through the questionnaire and did not do any other exercise program.

Increasing Participant Adherence to the Intervention: in order to increase participant adherence to the intervention, the research team adopted several strategies. First, the research team ensured that participants had a clear understanding of the purpose of the study, expected outcomes, and their own roles through detailed communication to enhance their sense of participation. Second, relevant education and training were provided to equip participants with the necessary exercise knowledge and skills to increase their confidence and motivation to participate. In addition, the research team encouraged participants to form support groups to enhance motivation to participate through mutual motivation and supervision. Regular feedback mechanisms were also key, as the research team showed participants their progress to enhance their sense of achievement while setting up small rewards to motivate continued participation. Flexible intervention scheduling took into account participants’ academic and life rhythms to minimize adherence problems caused by time conflicts. Finally, the research team maintains regular communication with participants to understand their feelings and challenges during the intervention process and promptly adjusts the measures to suit participants’ needs. Through these comprehensive measures, the research team aims to effectively improve participants’ adherence and ensure the smooth running of the study and the reliability of the data.

### 2.2. Sampling Method

The development of the study protocol and the implementation of the measurements were carried out by the following people:

The protocol of this study was developed by Yin-Ling Du, Kai Jiang, and Haojie Li. Yin-Ling Du was responsible for the overall study design and methodology to ensure the scientific validity of the study, while Kai Jiang was involved in the data collection and analysis to ensure the accuracy and consistency of the data during the study. Li focused on the implementation of respiratory measurements and data processing to ensure the standardization and reliability of the measurement process.

During the measurement process, the research team used cloth respiratory belts with embedded resistive stretch sensors to monitor participants’ respiratory rate and breathing movements. Specifically, two respiratory belts were used, one around the xiphoid process and the other around the navel, to observe both chest and abdominal breathing movements. Participants sat still for five minutes prior to the test to ensure a calm state and reduce the impact of abdominal circumference on the measurements. The research team played a neutral video during the test to distract the participant from their own breathing to ensure the accuracy of the measurements.

Data collection lasted 3 min, and the research team collected data between the 40th and 160th seconds after the start of the test to calculate the participant’s breathing pattern. All raw data was processed through the MATLAB App Designer program to calculate respiratory rate (RR), inspiratory duration (ID), and expiratory duration (ED). The research team ensured the reliability and validity of the measurements, providing a solid foundation for subsequent data analysis.

Physical education at Zhejiang University is a compulsory component of the curriculum, offering students the liberty to select from a diverse range of sports activities. These activities are grouped into six categories: (1) team sports such as football, basketball, and rugby; (2) racquet sports including badminton, table tennis, tennis, and volleyball; (3) combat sports such as taekwondo, boxing, and sanda; (4) performance sports comprising gymnastics, martial arts, and sports dancing; (5) cultural sports such as dragon boat and lion dance, kayaking; and (6) wellness activities including Tai Chi and swimming. With 474 physical education classes accommodating roughly 30 students each, the study aimed to sample 15 classes randomly—three from each activity group—to ensure a representative selection. Student recruitment proceeded sequentially within each category until 20 students of each gender had been recruited from each group, culminating in a total of 240 participants.

Randomization method

Establishment of the sample frame: the research team first determined the sample frame, which was 474 physical education courses at Zhejiang University. These courses covered six categories of sport activities, including team sports, racket sports, combat sports, performance sports, cultural sports and health activities.

Course selection: In order to ensure the representativeness of each type of activity, the research team randomly selected three courses from each sport category. Specifically, the process of random selection was as follows: using a random number generator (Excel), a random number was assigned to each course. Courses were ranked according to the generated random number, from which the top 3 courses were selected to ensure that each sport type was represented.

Student recruitment: Of the 15 courses selected, the research team recruited students sequentially. The recruitment process for each course was as follows: in the first class of each course, the researcher introduced students to the purpose, procedures, and participation requirements of the study.

After obtaining written informed consent from the students, the researchers recruited according to gender and age (18–22 years) until 20 male and 20 female participants were recruited in each sport category.

Randomized grouping: after recruitment was completed, all 240 participants were randomized into different experimental groups for subsequent testing and analysis. The process of random grouping was as follows: a random number was assigned to each participant using a random number generator.

Participants were sorted according to the random number and assigned to different experimental groups to ensure that each group was balanced in terms of gender and sport type.

Data Recording and Management: Throughout the randomization process, the research team maintained detailed records, including the selection of each course, the recruitment of participants, and the results of their randomization groups. These records helped to ensure the transparency and reproducibility of the study.

Test protocols and blinding
Test Protocol

The study used a series of standardized fitness tests to assess participants’ aerobic and anerobic exercise capacityanerobic exercise capacity. The tests included a 50 m dash, an 800 or 1000 m run (depending on gender), and a 12 min run. All tests were conducted on a standard athletic field to ensure environmental consistency. Prior to the test, participants are required to warm up sufficiently to prevent sports injuries and to enhance the effectiveness of the test. The process is as follows:

Warm-up: Participants perform a 10 min warm-up prior to the test, which includes dynamic stretching and light aerobic exercise to activate muscles and joints in various parts of the body.

50 m sprint: Participants performed the 50 m sprint test in order of number. Each participant wears a sensor band that is connected to a computerized system to ensure accurate timing. At the start of the test, participants prepare themselves at the starting line and start quickly when they hear the signal. Evaluators record each participant’s completion time to the nearest 0.1 s.

800 m or 1000 m run: Depending on gender, female participants performed the 800 m run and male participants performed the 1000 m run. Participants took their place at the starting line and started running on a signal, and the evaluators recorded the time to the nearest second through an electronic timing system. During the run, participants’ heart rate and respiratory rate were recorded in real time by monitoring equipment for subsequent analysis.

12 min run: The test was arranged on different weeks, and participants ran as far as possible within 12 min. During the test, a marker was set for every 100 m, and the assessor recorded the participant’s running distance at the end of each lap to ensure the accuracy of the data. Participants were required to report the distance to the nearest assessor immediately upon completion for recording purposes.

All assessors remained blind throughout the test to ensure the objectivity of the results. Participants’ group information was hidden prior to the test, and assessors recorded based on number only to avoid any potential bias.

Once data collection was complete, the research team organized and statistically analyzed all test results. Using statistical methods such as multiple regression analysis and K-means cluster analysis, the research team assessed the effects of different exercise programs on the participants’ exercise capacity and explored the correlation between breathing patterns and exercise performance. All data were analyzed by independent statisticians to ensure the reliability and scientific validity of the results.

In addition, the research team also considered the effects of participants’ body mass index (BMI) and abdominal exercises on exercise capacity in the data analysis, which showed that female participants’ lower BMI and higher proportion of abdominal exercises were positively correlated with their performance in the 800 m run. This finding provides an important reference for physical educators and healthcare professionals, emphasizing the potential of breathing pattern assessment to improve physical fitness and exercise capacity.

Blinded method

**Figure 1:**
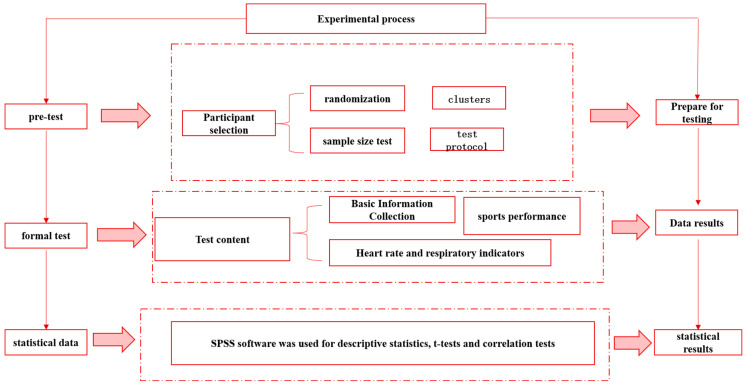
Sketch of the experimental flow.

In this study, blinded testing was used to ensure objectivity in outcome assessment and to minimize potential bias. During the running performance tests (e.g., 50 m sprint, 800 m run, and 12 min run), the assessors were blinded to the grouping information of the participants to ensure that the assessors could not identify the experimental or control group to which the participants belonged during the tests, thus avoiding any influence on the results of the tests due to the assessors’ expectations or biases. All participants were assigned a unique number by the research team for each participant prior to the running test to ensure that the assessors only recorded the results of the test without interfering or influencing the participant’s performance. During the data analysis phase, the research team separated the participants’ group information from the test results, and then correlated the results with the group information after the analysis was completed to ensure objectivity in the analysis. In addition, to further validate the effectiveness of the blind test, the research team used a variety of statistical methods (e.g., multiple regression analysis and K-means cluster analysis) in the analysis of the results and ensured that all results were analyzed by independent statisticians to further reduce the influence of subjective factors. Through these measures, this study effectively ensured blinded assessment of the results, thereby improving the reliability and scientific validity of the findings and enhancing the credibility of the study’s conclusions. Sketch of the experimental flow (Figure 1)

### 2.3. Inclusion and Exclusion

Participants were eligible if they were aged 18–22, free from musculoskeletal or cardiorespiratory conditions, females not in the menstrual period (days 3–10 post–menstruation), and willing to provide written informed consent. Exclusion criteria encompassed mental illness, recent or active acute illnesses without full recovery, consumption of caffeinated beverages within 2 h before testing, deviation in respiration or heart rate beyond ±2 standard deviations of their gender group’s mean, and inability to maintain stable respiratory cycles for 2 min. BMI over 30.

### 2.4. Respiratory Testing and Data Processing

**Figure 2:**
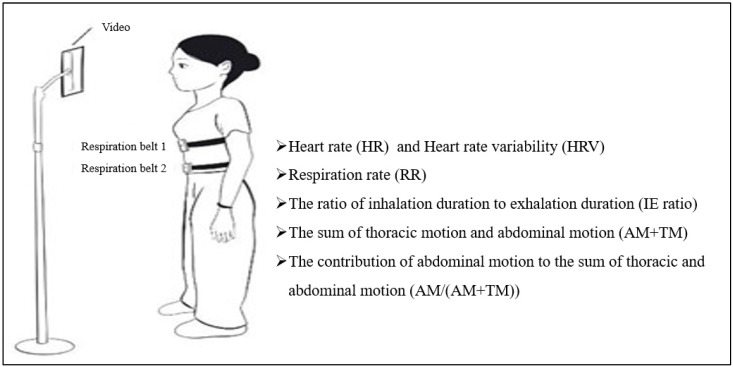
Test of spontaneous respiration and the list of relevant parameters.

Respiratory patterns were measured using fabric respiratory belts from Vernier (Beaverton, OR, USA), featuring embedded resistive stretch sensors to monitor respiration rate and breathing maneuvers. Two belts were utilized: one around the xiphoid process and another around the navel to simultaneously observe thoracic and abdominal movements. Participants were settled in a calm state for 5 min before testing and seated upright to minimize the impact of abdominal girth. The belts were adjusted to signal readiness with a green light, as per the user guide. To distract participants from their breathing during testing, a neutral video was played on an 11 inch Xiaomi Pad (resolution: 2560 × 1600; Xiaomi, Beijing, China), positioned 50–80 cm away. The testing period spanned 3 min, ensuring minimal intrusion and maximal comfort for. participants (Figure 2).

We observed that the respiration waves became more regular 30 s after the start of the test. Consequently, we took data from the 40th second to the 160th second (2 min in total) to calculate the breathing pattern. A MATLAB App Designer program (Matlab 2022a, Mathworks, Natick, MA, USA) was written to process these raw data and compute results.

**Figure 3:**
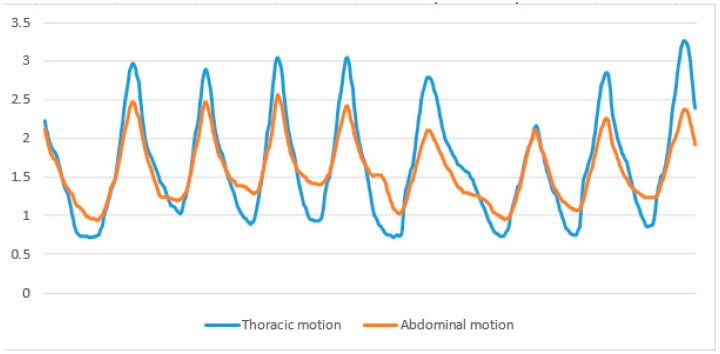
Wave lines representing respiration show the smoothed thoracic motion used to calculate respiration rate, inhalation duration, and exhalation duration.

To calculate the respiration rate (RR), inhalation duration (ID), and exhalation duration (ED), the Kernel distribution (smoothing) was computed using the formula for reducing the effect of noise. An h-value of 0.25 was empirically selected as the most appropriate. Then, code was written to find maximum values (peaks) where inspiration ended and minimum values (troughs) where expiration began. The inhalation duration (ID) was determined by subtracting the time of a peak from its preceding trough and subsequently obtaining an average from the cumulative IDs (Figure 3). Conversely, the exhalation duration (ED) was computed by subtracting the time of a trough from its preceding peak and averaging the totality of the accrued EDs. The I/E ratio was then derived by dividing ID by ED. The respiratory rate (per minute) was calculated by subtracting the time at which the first peak occurred from the time at which the last peak occurred. This result was then divided by the number of breaths taken in between to find the time for one breath. Finally, 60 was divided by the time taken for one breath.

**Figure 4:**
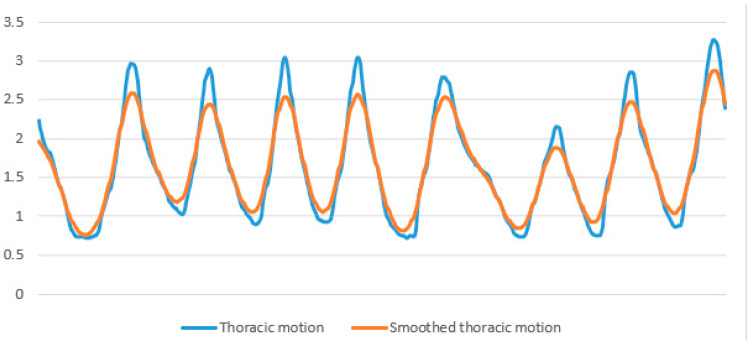
Displays wave lines of respiration, which represent raw data used for calculating thoracic motion and abdominal motion.

To measure the movements of the thoracic and abdominal regions, we analyzed the raw data. This approach was chosen because the noise present in the raw data does not impact the force values, unlike the use of smoothing techniques. We identified the peaks and troughs in the waveforms of each region (refer to Figure 4). The force of the thoracic movement was determined by subtracting the average of all trough values from the average of all peak values for thoracic movement. Abdominal movements were calculated using an identical approach.

### 2.5. Heart Rate and Heart Rate Variability Testing

**Figure 5:**
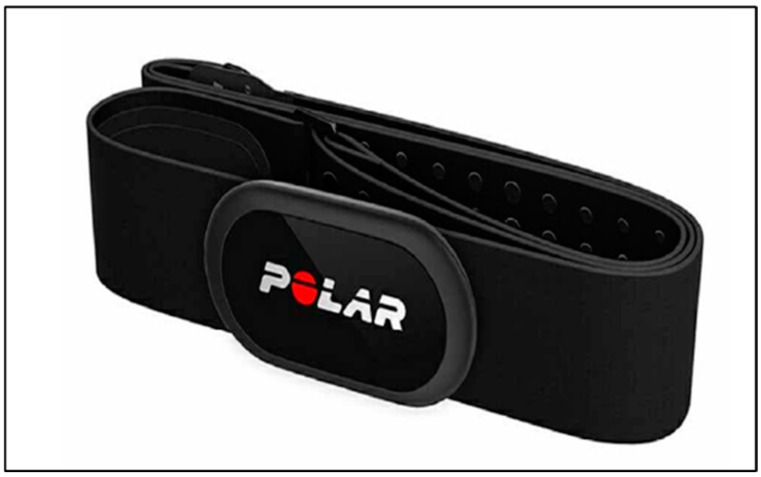
Polar heart rate sensor.

Following the resting heart rate test, the research team measured the participants’ heart rates over a six-minute period using a Polar H7 (refer to Figure 5) heart rate monitor (Kempele, Finland) to assess the students’ health status. The testing procedure was similar to the respiratory rate test, and participants remained seated during the test to ensure accuracy and consistency of the measurements. During the exercise testing phase, participants’ heart rates were also monitored in real time as they performed a 50 m sprint, a 1000 m run (for males) or an 800 m run (for females) and a 12 min run. Time domain metrics of heart rate variability (e.g., RMSSD) were further calculated from the heart rate data collected by the Polar H7 heart rate monitor, including resting heart rate and exercise center rate. Correlation analysis between these heart rate metrics and respiratory metrics provided a theoretical basis for understanding the relationship between respiratory metrics and exercise performance. With these data, the study was able to provide insight into how breathing patterns affect exercise performance.

### 2.6. Tests for Aerobic and Anerobic Exercise Capacities

To elucidate the connection between spontaneous respiratory patterns and aerobic and anerobic exercise capacities, this study utilized performances in the 50 m sprint, 1000 m run (for males)/800 m run (for females), and the 12 min run as proxies for anerobic capacity, mixed aerobic-anerobic capacity, and aerobic capacity, respectively. A quicker completion time in the 50 m sprint and the 1000 m/800 m run signifies stronger anerobic and mixed aerobic-anerobic capacities, whereas a greater distance covered in the 12 min run reflects a higher aerobic capacity.

The selection of these specific performance indicators is grounded in empirical evidence and established guidelines. Firstly, the 50 m sprint is acknowledged for its strong correlation with anerobic capacity [42]. Primarily utilizing the phosphagen system for its energy demands, a critical element of anerobic performance [43]. Secondly, the 1000 m (for males) and 800 m (for females) runs are recognized by the “National Student Physical Fitness and Health Standards” (Ministry of Education of China, 2014) as measures of mixed aerobic-anerobic capacity. These distances are understood to tax the aerobic and anerobic systems at proportions of 60/40% and 70/30% for men and women, respectively [44]. Thirdly, the 12 min run is a widely accepted measure for aerobic fitness [45], with a demonstrated correlation between distance covered and VO2max.

The evaluations were carried out on a standard athletic track. For the 50 m sprint and 800/1000 m runs, participants were equipped with sensor belts linked to a host computer by the instructor. Following a comprehensive warm-up, times for these runs were accurately recorded using an electronic timing system (Model: HK6800 B-WF, HENGKANGJIAYE Science and Technology Ltd., Shenzhen, China), featuring start and finish line sensors to ensure precision. The 50 m sprint was timed to the nearest 0.1 s, and for the gender-specific 800 m and 1000 m runs, to the nearest second. The 12 min run was scheduled for a different week, with the distance covered manually tracked. Markers were placed at 100 m intervals around the track, and officials recorded each lap completed by the participants. Uncertain results were verified using video recordings from four strategically placed cameras. Participants ran in groups, and upon the completion signal, they reported to the nearest official to confirm their distances, recorded to the nearest 100 m. This methodical approach ensured accurate and reliable measurement of each student’s running capacity.

### 2.7. Statistical Analysis

If the change in F-value was significant (*p* < 0.05), the relationship between the respiratory parameter and running performance was quadratic; otherwise, the relationship was regarded as linear, as previously described [46,47]. We employed a multiple regression analysis to investigate the relationship between respiratory patterns and running performance. The final model was determined using the adjusted coefficient of determination (R2) and statistical significance. To determine the statistical quality of the model, multicollinearity was verified by the variance inflation factor, and the homogeneity and normal distribution of the residuals were verified by graphic visual analysis. The level of significance was set at *p* < 0.05. To verify the results, we used the K-means clustering method to split each respiratory parameter into two or three groups according to the relationship of linearity and then compared the running ability between each group using univariate analysis of general linear models. The effect size was presented as partial eta squared (η^2^). The small effect size was 0.01, the medium effect size was 0.06, and the large effect size was 0.14.

## 3. Results

### 3.1. Data Description and Comparison between Men and Women

As shown in Table 1, there were significant differences in every respiration pattern parameter and running performance between male and female students. It should be noted that male students took the 1000 m run test, while females took the 800 m run test, so we did not compare their performances. Due to physical differences, we analyzed the data separately for each sex.

### 3.2. Correlation Test between RR and HRV-RMSSD

**Figure 6:**
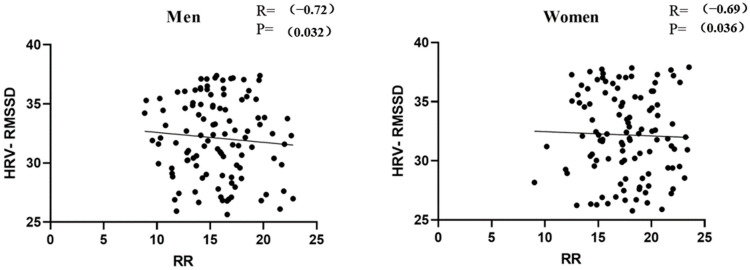
RR, respiration rate; s, second. HRV-RMSSD, heart rate variability-Root Mean Square of Successive Differences.

There is a significant negative correlation between RR interval and the RMSSD of heart rate variability (HRV) (Figure 6), implying that the higher the respiratory rate, the shorter the RR interval, and the lower the RMSSD value of HRV. This suggests that when the respiratory rate increases, the body’s sympathetic activity increases and parasympathetic activity decreases, which may result in the body being in a higher state of stress. Heart rate variability serves as an important physiological indicator of an individual’s autonomic nervous system function and recovery, which in turn affects exercise performance. Therefore, RR intervals, as a direct reflection of heart rate, can also indirectly reflect exercise performance, thus justifying our use of RR metrics for correlation analysis of exercise data.

### 3.3. The Linearity Assumption for the Relationship between Respiratory Patterns and Running Performance Is an Important Consideration in Research Studies

For the 50 m sprint time in male students, neither RR2, AM/(AM+TM)2, nor the I/E ratio2 significantly affected the F-value. However, the AM+TM2 treatment led to a significant increase in the F-value (*p* < 0.05), suggesting that a quadratic model better describes the relationship with AM+TM. Then, we observed a linear relationship between men’s 1000 m run time and 12 min run distance with each parameter of the respiratory pattern.

In female students, the 50 m sprint time had a quadratic relationship with RR and AM + TM, while the 12 min run distance had a quadratic relationship with RR. The remaining relationships between each respiratory pattern parameter and running performance were linear.

### 3.4. The Correlation between Respiratory Patterns and Running Performance

The multiple regression analysis for male participants revealed no significant correlation between 50 m sprint outcomes and factors such as age, BMI, RR, AM/(AM+TM), or the I/E ratio. Given the quadratic relationship between AM+TM and the 50 m sprint, AM+TM and its square were used as independent variables against the 50 m sprint time as the dependent variable. However, this model did not prove to be predictive (R^2^ = 0.046, ANOVA *p* = 0.083). Similarly, no significant correlations were identified for the 1000 m run with age, RR, AM+TM, AM/(AM+TM), or the I/E ratio. Consequently, the regression analysis did not find a meaningful relationship between men’s running performance and their respiratory patterns, and these results were omitted from the tables.

**Figure 7:**
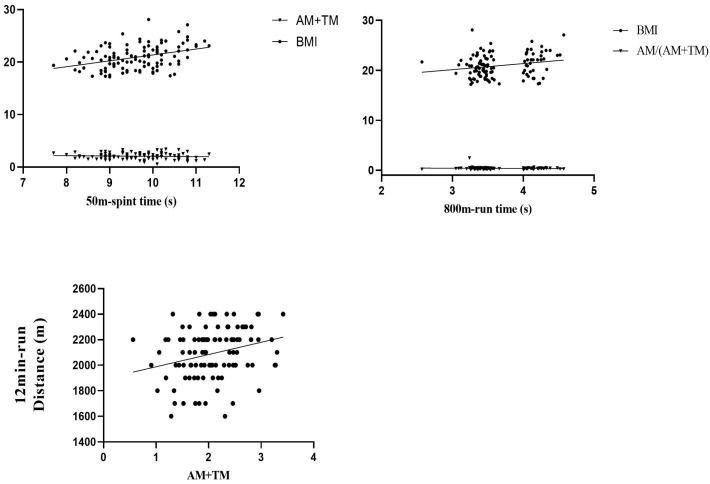
Scatterplot of correlation results.

For female students, as illustrated in Table 2 and Figure 7, the analysis addressed the quadratic relationship between AM+TM and 50 m sprint time, alongside a significant linear relationship with BMI. The multiple regression included age, BMI, AM+TM, and AM+TM^2^ as independent variables, with 50 m sprint time as the dependent outcome. This revealed that both BMI and AM+TM significantly impacted 50 m sprint times: higher BMI was associated with longer sprint times, whereas a mid-range AM+TM correlated with shorter times. In the 800 m run, a significant correlation was noted with BMI, and a linear relationship emerged with AM/(AM+TM). The analysis underscored that both BMI and AM/(AM+TM) significantly influenced 800 m run times, with higher BMI linked to longer durations and a higher AM/(AM+TM) associated with shorter times. Regarding the 12 min run, no significant correlation with age or BMI was found. However, a linear relationship with all respiratory pattern parameters was observed. A noteworthy positive correlation emerged between the 12 min run distance and AM+TM, indicating that a greater AM+TM is associated with a longer distance covered. No significant relationships were established between other respiratory parameters and running performance among women. This nuanced analysis underscores the complex interplay between respiratory patterns and physical performance, particularly highlighting gender-specific correlations.

### 3.5. Comparative Tests to Verify the Results of Correlation Tests

Students were classified into groups using K-means clustering according to their breathing patterns. Based on the relationship between each respiratory parameter and running performance, students were segmented into two groups for linear relationships and three for quadratic relationships. To normalize running performance data, residuals were created by correlating each running performance metric with BMI. The performance across these groups was then analyzed.

Given the quadratic relationship observed between AM+TM and 50 m sprint times, male students were categorized into three clusters based on their AM+TM values. A comparative analysis of 50 m sprint times across these clusters revealed no significant differences. Similarly, the times for the 800 m run and distances for the 12 min run showed no substantial variance between clusters differentiated by AM+TM values. For the respiration rate (RR) and the AM/(AM+TM) ratio, running performances did not significantly differ between the clusters. Nonetheless, the I/E ratio and its correlation with the 12 min run performance warrant additional investigation. Notably, there was a significant age difference between clusters formed based on I/E ratios. After accounting for age as a covariate, it was observed that men with lower I/E ratios achieved longer distances in the 12 min run, as detailed in Table 3. However, no significant differences were noted in the 50 m sprint and 1000 m run times between the clusters formed on the basis of the I/E ratio.

For female students, the 50 m sprint time was quadratically related to RR and AM+TM. Additionally, the 12 min run distance was also quadratically related to RR. The results showed that the three clusters had no significant differences in the 50 m sprint time based on RR and AM+TM, as well as no significant differences in the 12 min run distance based on RR. For the rest of the comparisons, we found that only the 12 min run distance was significantly longer for clusters with larger AM+TM, and the 800 m run time was significantly shorter for the cluster with larger AM/(AM+TM), as shown in Table 3. The remaining comparisons did not reveal any significant differences between the groups.

## 4. Discussion

The principal discovery of this investigation is that an increased combined abdominal and thoracic movement (AM+TM) forecasted a longer distance traversed in a 12 min run. Concurrently, a lower body mass index (BMI) paired with a greater ratio of abdominal movement to the sum of abdominal and thoracic movements (AM/(AM+TM)) correlated with reduced completion times in the 800 m run, a phenomenon observed solely in female participants.

Focusing on the association between AM+TM and the distance covered in the 12 min run, a Spearman’s correlation analysis revealed significant relationships for both AM (Rho = 0.245, *p* = 0.010) and TM (Rho = 0.206, *p* = 0.032) with the run distance. This suggests that AM and TM collectively play a role, resulting in a significant correlation between AM+TM and the 12 min run distance. Tidal volume refers to the amount of air that is inhaled or exhaled during normal breathing [48]. To some extent, the larger the AM+TM value, the deeper the breathing, and the corresponding tidal volume will be greater [49]. In general, adult males have about 500 milliliters and 400 milliliters for females [50]. During exercise, in order to obtain sufficient oxygen and reduce carbon dioxide, it is necessary to increase tidal volume to meet the demands of physical activity [51]. The 12 min run is mainly powered by aerobic energy, so we speculate that individuals with higher tidal volume during spontaneous breathing also have greater oxygen uptake during exercise. However, the correlation between the breathing amplitude during autonomous breathing and the breathing amplitude during a 12 min run is still unknown and requires further research.

Another significant observation is that females with a healthier BMI and a higher AM/(AM+TM) ratio showcased improved times in the 800 m run. Aligning with WHO standards, BMI is a crucial metric for assessing adult body fat [52]. Our results resonate with existing literature, indicating a positive correlation between BMI and 800 m run times [53]. Importantly, we found that AM/(AM+TM) might be another factor influencing the 800 m run time. Because AM/(AM+TM) is impacted by AM and TM, we assessed the effects of AM and TM separately. When controlling for BMI as a variable, the partial Spearman’s correlation results revealed a correlation of Rho = −0.161, *p* = 0.094 between AM and 800 m run time, and a correlation of Rho = −0.086, *p* = 0.372 between TM and 800 m run time. These results indicate that the primary factor influencing the correlation between AM/(AM+TM) and the 800 m run time is AM. From the perspective of energy metabolism, 800 m run belongs to middle-distance running that is highly dependent on the integrative contribution of both aerobic and anerobic energy systems [54,55]. Duffield et al. determined the aerobic/anerobic energy system contributions to 800 m running as 60/40% and 70/30% for men and women, respectively [40]. Based on this, we can infer that individuals with a larger abdominal contribution during spontaneous breathing had a better aerobic and anerobic mixed capacity. The diaphragm and external intercostal muscles are the major inhalation muscles [56]. When the diaphragm contracts, it flattens, and the dome descends, thus increasing the vertical length of the chest cavity, which decrease in the intrathoracic pressure enables air to enter the lungs [57]. Along with the contraction of the diaphragm, it pushes the internal organs down and expands the abdominal wall. Therefore, a larger AM/(AM+TM) ratio represents a larger diaphragmatic motion. The descent of the diaphragm causes a decrease in chest pressure, and the alveoli at the bottom of the lungs are fully opened. Meanwhile, due to the effect of gravity, the blood flow at the bottom of the lungs is greater than that at the top, making it more conducive to gas exchange. In fact, ventilation is 50% greater at the base of the lung than at the apex [58], and diaphragmatic breathing can improve ventilation distribution [59], maximal exercise tolerance, and oxygen uptake [60,61]. This can provide sufficient oxygen for the 800 m run. At the same time, the diaphragm can effectively maintain the stability of the trunk [62]. This can help us maintain a good running posture, improve balance, and reduce oxygen consumption while running [38,63]. Therefore, we can predict that subjects with a higher proportion of abdominal breathing under autonomous breathing tend to have better performance in 800 m running. This may provide an indicator for predicting women’s middle-distance race.

Our research further revealed that abdominal movement’s contribution to performance in the 1000 m run was not significantly correlated for males, possibly due to differing respiratory patterns between genders. Historical observations by Dr. Hutchinson in 1846 highlighted distinct breathing styles in men and women: men primarily utilize diaphragmatic breathing, whereas women predominantly employ thoracic breathing [64]. This distinction becomes especially noticeable in seated positions and under various postural changes [65,66,67]. That is to say, women tend to engage in thoracic breathing, while men are more inclined to abdominal breathing. The underpinning reasons for these gender-specific breathing patterns are largely anatomical and hormonal [68,69]. Women, for instance, typically possess a narrower chest cavity [64], shorter ribs [70], and a shorter, higher-positioned diaphragm compared to men [71]. Additionally, women generally have lower trunk muscle mass. Given these differences, it’s plausible that abdominal movements might exert a more pronounced effect on women’s respiratory efficiency and, by extension, their performance in activities requiring substantial oxygen intake, such as the 800 m run. Conversely, the constraints imposed by a lesser emphasis on chest expansion during respiration might influence women’s physiological responses during exertion differently.

This gender disparity in breathing strategies bears significant implications for sports training and health promotion. Recognizing and addressing these differences can aid athletes, coaches, and fitness enthusiasts in optimizing training regimens and enhancing overall physical well-being.

**Applied value of the study to learning**: this study examines the relationship between spontaneous breathing patterns and athletic performance in a population of college students, emphasizing the practical application of learning with far-reaching educational implications. First, college students are at a critical stage of physical development and ability enhancement and understanding how breathing patterns affect athletic performance can help them train more effectively in physical education programs. By mastering scientific breathing techniques, students can improve their endurance and speed in aerobic exercises such as running and swimming and thus perform better in class and extracurricular activities. Second, the results of the study provide valuable teaching resources for physical education. Teachers can incorporate respiratory training into their curriculum to help students recognize the close relationship between breathing and athletic performance. Through practical activities, students can experience firsthand the effects of different breathing patterns on exercise performance, enhancing their understanding of bodily functions. This combination of theoretical and practical learning not only improves students’ motor skills but also develops their scientific thinking and independent learning ability. In addition, the study encourages students to pay attention to their physical fitness and health management. By learning how to optimize breathing patterns, students can better manage stress and anxiety in their daily lives and improve their overall mental health. This cultivation of health awareness not only helps them in their studies and lives during school but also lays the foundation for their future careers and lifestyles. In summary, the study not only provides college students with a scientific basis for improving their athletic ability but also emphasizes the practical application of learning in physical education. By integrating the research results into the curriculum and daily training, students are able to better understand and apply exercise science, promote the overall improvement of physical fitness, and cultivate lifelong exercise habits. This type of learning not only contributes to students’ academic development but also supports their healthy lives and future careers.

**Limitations of the study**: limitations of the study include the small sample size, limited to 240 college students, which may affect the generalizability of the results. In addition, the strict exclusion criteria may have led to sample selection bias, limiting the external validity of the study. The accuracy of the respiratory measurements may also have been compromised, failing to fully capture respiratory changes in participants during different exercise states. In addition, the study was only conducted in a specific setting and did not consider the potential impact of environmental factors on the results. These limitations suggest that future studies need to expand the sample size and consider additional variables.

## 5. Conclusions

Female college students exhibiting a higher tidal volume, represented by the combined thoracic and abdominal movements during spontaneous breathing, could experience enhanced oxygen uptake during physical activity. Furthermore, when adjusting for body size (BMI), an increase in abdominal movements might enhance their overall aerobic and anerobic capacities.

## Figures and Tables

**Table 1:** Data description and comparison between men and women.

	Men		Women				
All Parameters	n	Mean ± SD	n	Mean ± SD	F	*p*	η^2^
Age (year)	109	19.8 ± 0.82	110	19.9 ± 0.78	0.19	0.665	0.001
Height (cm)	109	174 ± 6.28	110	162 ± 5.24	224	<0.001	0.508
Weight (kg)	109	65.1 ± 8.88	110	55.1 ± 6.78	88.1	<0.001	0.289
BMI (kg/m^2^)	109	21.5 ± 2.41	110	20.9 ± 2.28	3.11	0.079	0.014
HR (rep/min)	109	84.7 ± 10.3	110	89.8 ± 10.4	12.9	<0.001	0.056
HRV-RMSSD (ms)	109	31.31 ± 6.18	110	31.50 ± 6.42	9.13	0.456	0.013
RR (rep/min)	109	15.7 ± 3.22	110	17.5 ± 3.17	18.5	<0.001	0.079
AM+TM (N)	109	3.69 ± 1.49	110	2.08 ± 0.56	112	<0.001	0.341
AM/(AM+TM) (N)	109	0.41 ± 0.14	110	0.35 ± 0.11	11.6	0.001	0.051
IE ratio	109	0.84 ± 0.11	110	0.78 ± 0.11	15.9	<0.001	0.068
50 m sprint time (s)	109	7.86 ± 0.55	110	9.62 ± 0.76	385	<0.001	0.640
800 m run time (min)			110	3.66 ± 0.40			
1000 m run time (min)	109	4.13 ± 0.41					
12 min run distance (m)	109	2548 ± 228	109	2092 ± 197	250	<0.001	0.537

Notes: AM+TM, abdominal motion plus thoracic motion; AM/(AM+TM), proportion of abdominal motion to the sum of abdominal and thoracic motions; BMI, body mass index; HR, heart rate; I/E ratio, ratio of inhalation duration to exhalation duration; m, meter; min, minute; N, Newton; RR, respiration rate; s, second. HRV-RMSSD, heart rate variability-Root Mean Square of Successive Differences.

**Table 2:** The correlation between respiratory patterns and running performance.

	Dependent Variables	Independent Variables	B	95% CI	β	*p^β^*	R^2^	F	*p^F^*
Women	50 m sprint time (s)	BMI	0.107	0.047–0.168	0.321	<0.001	0.189	6.098	<0.001
		AM+TM	−1.351	−2.700–−0.003	−0.993	0.050			
	800 m run time (s)	BMI	0.041	0.009–0.074	0.234	0.013	0.083	4.869	0.009
		AM/(AM+TM)	−0.722	−1.410–−0.035	−0.194	0.040			
	12 min run Distance (m)	AM+TM	96.659	32.383–161.817	0.276	0.004	0.076	8.847	0.004

Notes: AM+TM, abdominal motion plus thoracic motion; AM/(AM+TM), proportion of abdominal motion to the sum of abdominal and thoracic motions; BMI, body mass index; s, second; m, meter; *p*^β^ is the *p* value of β, *p^F^* is the *p* value from F. Group 1 refers to participants with lower respiratory parameters, and Group 2 refers to participants with higher respiratory parameters.

**Table 3:** The Comparison of Running Performance in Lower/Higher Respiratory Parameter Groups.

Sex	Respiratory Patterns and Running Performance	Group 1		Group 2		F	*p*	η^2^
		n	Mean ± SD	n	Mean ± SD			
Men	I/E ratio	41	0.73 ± 0.06	68	0.90 ± 0.06	185	<0.001	0.63
	12 min run distance residuals		56.4 ± 210		−34.0 ± 223	4.41	0.038	0.04
Women	AM+TM	58	1.66 ± 0.33	51	2.56 ± 0.34	194	<0.001	0.64
	12 min run distance residuals		−39.1 ± 192		44.4 ± 181	5.42	0.022	0.05
	AM/(AM+TM)	54	0.44 ± 0.06	56	0.27 ± 0.07	206	<0.001	0.66
	800 m run time residuals		−0.08 ± 0.38		0.08 ± 0.40	4.589	0.034	0.04

Notes: AM+TM, abdominal motion plus thoracic motion; AM/(AM+TM), proportion of abdominal motion to AM+TM; I/E ratio, inhalation/exhalation ratio.

## Data Availability

The data that support the findings of this study are available on request from the corresponding author.

## References

[1] Foreman K.J., Marquez N., Dolgert A., Fukutaki K., Fullman N., McGaughey M., Pletcher M.A., Smith A.E., Tang K., Yuan C.-W. (2018). Forecasting Life Expectancy, Years of Life Lost, and All-Cause and Cause-Specific Mortality for 250 Causes of Death: Reference and Alternative Scenarios for 2016-40 for 195 Countries and Territories. Lancet.

[2] Ford E.S., Giles W.H., Dietz W.H. (2002). Prevalence of the Metabolic Syndrome among US Adults: Findings from the Third National Health and Nutrition Examination Survey. JAMA.

[3] Stefan N., Schulze M.B. (2023). Metabolic Health and Cardiometabolic Risk Clusters: Implications for Prediction, Prevention, and Treatment. Lancet Diabetes Endocrinol..

[4] Rognmo Ø., Hetland E., Helgerud J., Hoff J., Slørdahl S.A. (2004). High Intensity Aerobic Interval Exercise Is Superior to Moderate Intensity Exercise for Increasing Aerobic Capacity in Patients with Coronary Artery Disease. Eur. J. Cardiovasc. Prev. Rehabil..

[5] Aerobic vs. Anaerobic Exercise Training Effects on the Cardiovascular System. https://read.qxmd.com/read/28289526/aerobic-vs-anaerobic-exercise-training-effects-on-the-cardiovascular-system.

[6] Ferguson B. (2014). ACSM’s Guidelines for Exercise Testing and Prescription.

[7] Garber C.E., Blissmer B., Deschenes M.R., Franklin B.A., Lamonte M.J., Lee I.-M., Nieman D.C., Swain D.P., American College of Sports Medicine American College of Sports Medicine Position Stand (2011). Quantity and Quality of Exercise for Developing and Maintaining Cardiorespiratory, Musculoskeletal, and Neuromotor Fitness in Apparently Healthy Adults: Guidance for Prescribing Exercise. Med. Sci. Sports Exerc..

[8] Warburton D.E.R., Nicol C.W., Bredin S.S.D. (2006). Health Benefits of Physical Activity: The Evidence. CMAJ.

[9] Haskell W.L., Lee I.-M., Pate R.R., Powell K.E., Blair S.N., Franklin B.A., Macera C.A., Heath G.W., Thompson P.D., Bauman A. (2007). Physical Activity and Public Health: Updated Recommendation for Adults from the American College of Sports Medicine and the American Heart Association. Med. Sci. Sports Exerc..

[10] Carnethon M.R., Gidding S.S., Nehgme R., Sidney S., Jacobs D.R., Liu K. (2003). Cardiorespiratory Fitness in Young Adulthood and the Development of Cardiovascular Disease Risk Factors. JAMA.

[11] Benito-Ruiz P., Camacho-Zambrano M.M., Carrillo-Arcentales J.N., Mestanza-Peralta M.A., Vallejo-Flores C.A., Vargas-López S.V., Villacís-Tamayo R.A., Zurita-Gavilanes L.A. (2009). A Randomized Controlled Trial on the Efficacy and Safety of a Food Ingredient, Collagen Hydrolysate, for Improving Joint Comfort. Int. J. Food Sci. Nutr..

[12] Laukkanen J.A., Rauramaa R., Salonen J.T., Kurl S. (2007). The Predictive Value of Cardiorespiratory Fitness Combined with Coronary Risk Evaluation and the Risk of Cardiovascular and All-Cause Death. J. Intern. Med..

[13] Peak Aerobic Capacity Predicts Prognosis in Patients with Coronary Heart Disease—PubMed. https://pubmed.ncbi.nlm.nih.gov/18657659/.

[14] Prevalence, Phenotype and Cardiometabolic Risk of Polycystic Ovary Syndrome under Different Diagnostic Criteria—PubMed. https://pubmed.ncbi.nlm.nih.gov/22777527/.

[15] The Blood Lactate Response to Exercise|Semantic Scholar. https://www.semanticscholar.org/paper/The-blood-lactate-response-to-exercise-Weltman/ce6415e100a2971ef02dc53b3c3303e8f5f042cd.

[16] Schoenfeld B.J. (2010). The Mechanisms of Muscle Hypertrophy and Their Application to Resistance Training. J. Strength Cond. Res..

[17] Fundamentals of Resistance Training: Progression and Exercise Prescription—PubMed. https://pubmed.ncbi.nlm.nih.gov/15064596/.

[18] Parkes R. Rate of Respiration: The Forgotten Vital Sign. https://journals.rcni.com/emergency-nurse/rate-of-respiration-the-forgotten-vital-sign-en2011.05.19.2.12.c8504.

[19] Paz J.C., West M.P. (2019). Acute Care Handbook for Physical Therapists.

[20] (PDF) Pre-Exercise Hyperventilation Can Significantly Increase Performance in the 50-Meter Front Crawl. https://www.researchgate.net/publication/275671458_Pre-exercise_hyperventilation_can_significantly_increase_performance_in_the_50-meter_front_crawl.

[21] Hyperventilation as a Strategy for Improved Repeated Sprint: The Journal of Strength & Conditioning Research. https://journals.lww.com/nsca-jscr/Fulltext/2014/04000/Hyperventilation_as_a_Strategy_for_Improved.33.aspx.

[22] Wallin B.G., Hart E.C., Wehrwein E.A., Charkoudian N., Joyner M.J. (2010). Relationship between Breathing and Cardiovascular Function at Rest: Sex-Related Differences. Acta Physiol..

[23] Russo M.A., Santarelli D.M., O’Rourke D. (2017). The Physiological Effects of Slow Breathing in the Healthy Human. Breathe.

[24] Migliaccio G.M., Russo L., Maric M., Padulo J. (2023). Sports Performance and Breathing Rate: What Is the Connection? A Narrative Review on Breathing Strategies. Sports.

[25] Ragnarsdóttir M., Kristinsdóttir E.K. (2006). Breathing Movements and Breathing Patterns among Healthy Men and Women 20–69 Years of Age. Reference Values. Respiration.

[26] Bae D., Matthews J.J.L., Chen J.J., Mah L. (2021). Increased Exhalation to Inhalation Ratio during Breathing Enhances High-Frequency Heart Rate Variability in Healthy Adults. Psychophysiology.

[27] Van Diest I., Verstappen K., Aubert A.E., Widjaja D., Vansteenwegen D., Vlemincx E. (2014). Inhalation/Exhalation Ratio Modulates the Effect of Slow Breathing on Heart Rate Variability and Relaxation. Appl. Psychophysiol. Biofeedback.

[28] Cenk Gürkan A., Söyler M., Subak E. (2022). Efectos de diez semanas de entrenamiento funcional en niños de 8-10 años sobre parámetros respiratorios y motores. Apunt. Univ..

[29] Collins E.G., Jelinek C., O’Connell S., Butler J., McBurney C., Gozali C., Reda D., Laghi F. (2014). Contrasting breathing retraining and helium–oxygen during pulmonary rehabilitation in COPD: A randomized clinical trial. Respir. Med..

[30] Li Z., Li W., Lin P., Jia T., Ji L., Li C. (2023). Motor-Respiratory Coupling Improves Endurance Performance during Rhythmic Isometric Handgrip Exercise. Med. Sci. Sports Exerc..

[31] Csepregi É., Szekanecz Z., Szántó S. (2020). The effects of breathing exercises in comparison with other exercise programs on cardiorespiratory fitness among healthy female college students. J. Sports Med. Phys. Fit..

[32] Harbour E., Lasshofer M., Genitrini M., Schwameder H. (2021). Enhanced Breathing Pattern Detection during Running Using Wearable Sensors. Sensors.

[33] Wang R., Yu Y., Zuo Y., Gu Z., Li H., Chen H., Lu W. (2024). Airflow-Induced Flexoelectric Bending Sensors for Human Breath Detection. IEEE Sens. J..

[34] Das P.S., Ahmed H.E.U., Motaghedi F., Lester N.J., Khalil A., Janaideh M.A., Anees S., Carmichael T.B., Bain A.R. (2023). A Wearable Multisensor Patch for Breathing Pattern Recognition. IEEE Sens. J..

[35] Chen R., Formenti F., Obeid A., Hahn C.E.W., Farmery A.D. (2013). A fibre-optic oxygen sensor for monitoring human breathing. Physiol. Meas..

[36] Diaphragmatic Breathing vs. Chest Breathing. https://www.lifelongwellness.org/wellness-academy/health/diaphragmatic-breathing-vs-chest-breathing/.

[37] Figueroba A. Types of Breathing: Diaphragmatic, Thoracic, Clavicular. https://healthywaymag.com/psychology/types-of-breathing.

[38] (PDF) Relationship of Breathing Exercises with Improvement of Postural Stability in Healthy Adults. https://www.researchgate.net/publication/320196403_RELATIONSHIP_OF_BREATHING_EXERCISES_WITH_IMPROVEMENT_OF_POSTURAL_STABILITY_IN_HEALTHY_ADULTS.

[39] Kocjan J., Gzik-Zroska B., Nowakowska K., Burkacki M., Suchoń S., Michnik R., Czyżewski D., Adamek M. (2018). Impact of Diaphragm Function Parameters on Balance Maintenance. PLoS ONE.

[40] Barbosa A., Martins F., Silva A., Coelho A., Intelangelo L., Vieira E. (2017). Activity of Lower Limb Muscles During Squat with and Without Abdominal Drawing-In and Pilates Breathing. J. Strength Cond. Res..

[41] Liang W.-M., Bai Z.-M., Aihemaiti M., Yuan L., Hong Z.-M., Xiao J., Ren F.-F., Rukšėnas O. (2022). Women’s Respiratory Movements during Spontaneous Breathing and Physical Fitness: A Cross-Sectional, Correlational Study. Int. J. Environ. Res. Public Health.

[42] Dardouri W., Selmi M.A., Sassi R.H., Gharbi Z., Rebhi A., Yahmed M.H., Moalla W. (2014). Relationship Between Repeated Sprint Performance and Both Aerobic and Anaerobic Fitness. J. Hum. Kinet..

[43] Sinnett A.M., Berg K., Latin R.W., Noble J.M. (2001). The Relationship between Field Tests of Anaerobic Power and 10-Km Run Performance. J. Strength Cond. Res..

[44] Duffield R., Dawson B., Goodman C. (2005). Energy System Contribution to 400-Metre and 800-Metre Track Running. J. Sports Sci..

[45] McCutcheon M.C., Sticha S.A., Giese M.D., Nagle F.J. (1990). A Further Analysis of the 12-Minute Run Prediction of Maximal Aerobic Power. Res. Q. Exerc. Sport.

[46] Abdelmageed R.I., Elhenawy Y.I., Zaafar D.K., Abdelaziz A.W. (2022). Coping Strategies among Children and Adolescents: Validity and Reliability of the Arabic Version of the Kidcope Scale. Heliyon.

[47] Mascret N., Nicolleau M., Ragot-Court I. (2020). Development and Validation of a Scale Assessing Achievement Goals in Driving. PLoS ONE.

[48] Matheus G.B., Dragosavac D., Trevisan P., Costa C.E.d., Lopes M.M., Ribeiro G.C.d.A. (2012). Inspiratory Muscle Training Improves Tidal Volume and Vital Capacity after CABG Surgery. Rev. Bras. Cir. Cardiovasc..

[49] Duffty P., Spriet L., Bryan M.H., Bryan A.C. (1981). Respiratory Induction Plethysmography (Respitrace): An Evaluation of Its Use in the Infant. Am. Rev. Respir. Dis..

[50] Hallett S., Toro F., Ashurst J.V. (2024). Physiology, Tidal Volume.

[51] Pierce R. (2005). Spirometry: An Essential Clinical Measurement. Aust. Fam. Physician.

[52] Research on the Correlation between Body Mass Index and Physical Health Index of Medical College Students. https://www.researchgate.net/publication/354558683_Research_on_the_Correlation_between_Body_Mass_Index_and_Physical_Health_Index_of_Medical_College_Students.

[53] Chen X., Cui J., Zhang Y., Peng W. (2020). The Association between BMI and Health-Related Physical Fitness among Chinese College Students: A Cross-Sectional Study. BMC Public Health.

[54] Brandon L.J., Boileau R.A. (1992). Influence of Metabolic, Mechanical and Physique Variables on Middle Distance Running. J. Sports Med. Phys. Fit..

[55] Lacour J.R., Padilla-Magunacelaya S., Barthélémy J.C., Dormois D. (1990). The Energetics of Middle-Distance Running. Eur. J. Appl. Physiol..

[56] Higashino M., Miyata K., Kudo K. (2022). Coordination Dynamics of Thoracic and Abdominal Movements during Voluntary Breathing. Sci. Rep..

[57] De Troyer A., Boriek A.M. (2011). Mechanics of the Respiratory Muscles. Compr. Physiol..

[58] Powers K.A., Dhamoon A.S. (2024). Physiology, Pulmonary Ventilation and Perfusion.

[59] Vitacca M., Clini E., Bianchi L., Ambrosino N. (1998). Acute Effects of Deep Diaphragmatic Breathing in COPD Patients with Chronic Respiratory Insufficiency. Eur. Respir. J..

[60] Ambrosino N., Paggiaro P.L., Macchi M., Filieri M., Toma G., Lombardi F.A., Del Cesta F., Parlanti A., Loi A.M., Baschieri L. (1981). A Study of Short-Term Effect of Rehabilitative Therapy in Chronic Obstructive Pulmonary Disease. Respiration.

[61] Strunk R.C., Mascia A.V., Lipkowitz M.A., Wolf S.I. (1991). Rehabilitation of a Patient with Asthma in the Outpatient Setting. J. Allergy Clin. Immunol..

[62] Huxel Bliven K.C., Anderson B.E. (2013). Core Stability Training for Injury Prevention. Sports Health.

[63] Byars A., Gandy-Moodie N., Greenwood L., Stanford M.S., Greenwood M. (2011). An Evaluation of the Relationships between Core Stability, Core Strength, and Running Economy in Trained Runners. J. Strength Cond. Res..

[64] On the Capacity of the Lungs, and on the Respiratory Functions, with a View of Establishing a Precise and Easy Method of Detecting Disease by the Spirometer—Abstract—Europe PMC. https://europepmc.org/article/MED/20895846.

[65] Verschakelen J.A., Demedts M.G. (1995). Normal Thoracoabdominal Motions. Influence of Sex, Age, Posture, and Breath Size. Am. J. Respir. Crit. Care Med..

[66] Gilbert R., Auchincloss J.H., Peppi D. (1981). Relationship of Rib Cage and Abdomen Motion to Diaphragm Function during Quiet Breathing. Chest.

[67] Binazzi B., Lanini B., Bianchi R., Romagnoli I., Nerini M., Gigliotti F., Duranti R., Milic-Emili J., Scano G. (2006). Breathing Pattern and Kinematics in Normal Subjects during Speech, Singing and Loud Whispering. Acta Physiol..

[68] Bellemare F., Jeanneret A., Couture J. (2003). Sex Differences in Thoracic Dimensions and Configuration. Am. J. Respir. Crit. Care Med..

[69] LoMauro A., Aliverti A. (2015). Respiratory Physiology of Pregnancy. Breathe.

[70] LoMauro A., Aliverti A. (2018). Sex Differences in Respiratory Function. Breathe.

[71] Johannesdottir F., Allaire B., Anderson D.E., Samelson E.J., Kiel D.P., Bouxsein M.L. (2018). Population-Based Study of Age- and Sex-Related Differences in Muscle Density and Size in Thoracic and Lumbar Spine: The Framingham Study. Osteoporos. Int..

